# The Intraoperative Use of a Portable Cone-Beam Computed Tomography System for the Diagnosis of Intraperitoneal Bladder Perforation

**DOI:** 10.1155/2021/2060572

**Published:** 2021-09-23

**Authors:** Ankur Choksi, Benjamin Press, Cayce Nawaf, Shannon Longyear, Marc Ferrante, Thomas V. Martin

**Affiliations:** ^1^Department of Urology, Yale University School of Medicine, New Haven, CT, USA; ^2^Department of Radiology and Biomedical Imaging, Yale University School of Medicine, New Haven, CT, USA

## Abstract

**Background:**

Intraoperative imaging for endourologic procedures is generally limited to single-plane fluoroscopic X-ray. The O-arm™ is a mobile cone-bean CT scanner that may have applications in urologic surgeries. *Case Presentation*. We present a case of an 85-year-old male with radiation cystitis and recurrent gross hematuria who was identified to have a bladder perforation on cystoscopy during emergent clot evacuation. Single-view fluoroscopic evaluation was inconclusive as to whether an intraperitoneal bladder perforation occurred. A portable cone-beam CT scan was used to acquire a 3-D CT cystogram, which demonstrated intraperitoneal contrast extravasation, confirming the diagnosis of an intraperitoneal bladder perforation.

**Conclusion:**

We report the first use of a portable cone-beam CT scanner to perform an intraoperative CT cystogram to diagnose an intraperitoneal bladder perforation and guide surgical management.

## 1. Introduction

Cone-beam computed tomography (CBCT) is an increasingly available mobile imaging modality that can be used for intraoperative planning. The O-arm™ is a mobile imaging system that produces cone-beam X-rays in a rotating tube, capable of fitting around the surgical table, and can acquire 2-D fluoroscopic images and rapid 3-D volumetric reconstruction. The portability of a CBCT scanner, such as the O-arm™, allows for real-time intraoperative imaging and decision making, eliminating the need to transport the patient to a traditional CT scanner while the patient is under general anesthesia. Additional benefits of CBCT include lower radiation exposure to the patient compared to conventional CT, and superior spatial resolution and multidimensional image acquisitions when compared to fluoroscopy [1]. At our institution, the application of intraoperative CBCT is well established for neurosurgical, spinal, and orthopedic surgery. For urologic surgeries, intraoperative imaging is obtained through anterior-posterior plane 2-D fluoroscopic imaging.

For the diagnosis of bladder perforation, cystography with the use of water-soluble contrast is acquired either through fluoroscopy or CT imaging [2]. Cystography also allows for the determination as to whether the bladder perforation has occurred intraperitoneal or extraperitoneal, which would guide further management. If fluoroscopy is used, at least an anterior-posterior and lateral view must be acquired. For simple extraperitoneal bladder perforations, management may be limited to an indwelling urinary catheter. For intraperitoneal bladder perforations, tracking of the urine into the peritoneal cavity can cause ileus, peritonitis, sepsis, and abdominal compartment syndrome and is unlikely to heal with urinary drainage; hence, operative repair is warranted. For an intraoperative detection of bladder perforation visualized on rigid cystoscopy, a retrograde cystogram with single-plane fluoroscopy has limited utility due to the inability to obtain lateral plane views. Herein, we present the first reported utilization of a portable conical-beam CT scanner to perform an intraoperative CT cystogram to assist in the differentiation between an intraperitoneal and extraperitoneal bladder perforation visualized on cystoscopy.

## 2. Case Presentation

### 2.1. Clinical History

The patient is an 85-year-old male with a remote history of prostate cancer treated with an open radical prostatectomy and adjuvant radiation therapy who subsequently developed recurrent gross hematuria and clot retention secondary to radiation cystitis. He completed approximately forty sessions of hyperbaric oxygen therapies but continued to experience recurrent gross hematuria requiring multiple inpatient hospitalizations for continuous bladder irrigations and requiring transfusion of blood products. He was discharged from the hospital two weeks prior to his most recent admission with a urinary catheter after a prolonged hospital course during which he was treated for gross hematuria. His hospital course was also complicated by a small bowel obstruction managed nonoperatively and a *C. difficile* infection. On the night prior to this admission, he noticed poor drainage from his urinary catheter prompting his presentation to an outside Emergency Department where he was initiated on continuous bladder irrigation and transferred to our institution.

**Figure 1:**
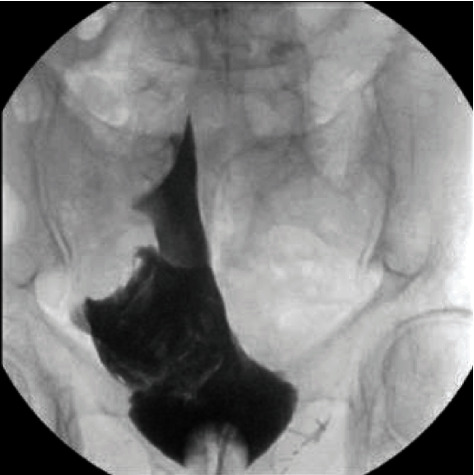
Intraoperative fluoroscopic cystography with images captured in the anterior-posterior view. Fluoroscopic images demonstrate the bladder perforation; however, with a single view, it is unclear whether the extravasation of contrast is intraperitoneal.

The following morning, the patient endorsed diffuse lower abdominal pain, had a marked increase in his leukocytosis, and had a poorly draining catheter with worsening gross hematuria. The patient was brought to the operating room emergently for a cystoscopy and clot evacuation. After clot was removed from the bladder and better visualization was obtained, a perforation was visualized at the dome of the bladder that extended towards the base of the bladder. In order to differentiate between an extraperitoneal and intraperitoneal bladder perforation, a fluoroscopic retrograde cystogram limited to the anterior-posterior view in lithotomy position was performed (Figure 1) using approximately 50 milliliters of iohexol (Omnipaque™) contrast dye; however, the image findings were equivocal.

### 2.2. Technique

**Figure 2:**
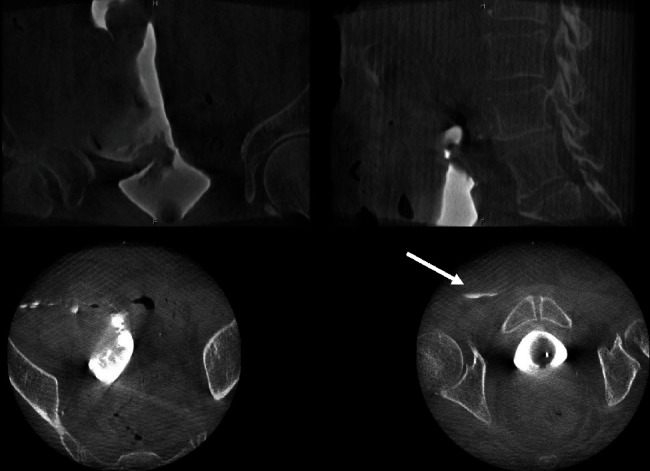
Intraoperative CT cystogram obtained using an O-arm™ surgical imaging system with three-dimensional volumetric reconstruction. Image acquisition in the coronal (top left), sagittal (top right), axial abdomen (bottom left), and axial pelvis (bottom right) planes was obtained. Contrast is visible in the intraperitoneal cavity and around the bowel, confirming the diagnosis of an intraperitoneal bladder perforation. Note the extravasation of contrast into a right inguinal hernia in the axial pelvis plane, also confirming an intraperitoneal bladder perforation.

A portable cone-beam CT scanner (O-arm™, Medtronic, Minneapolis, MN) was brought into the operating room and positioned around the surgical table. An additional 50 milliliters of Omnipaque™ contrast was instilled by gravity drainage into the bladder. Two-dimensional fluoroscopic images and three-dimensional conical-beam computed tomography (CBCT) images of the abdomen and pelvis were obtained (Figure 2), to represent an intraoperative CT cystogram. Contrast extravasation was visualized in the peritoneal cavity. After the images were reviewed by radiology, it was confirmed that the patient had an intraperitoneal bladder perforation. The patient was repositioned and prepped and underwent an exploratory laparotomy and repair of his intraperitoneal bladder perforation. The total fluoroscopy radiation exposure from the O-arm™ was 9.23 mGy over an irradiation duration of 5.43 seconds. For the 3-D CBCT acquisition at 120 kVP, the CT dose index was 27.47 mGy with a dose-length product of 439.39 mGy∗cm.

## 3. Discussion

We described the first reported use of an intraoperative CBCT cystogram for the diagnosis of an intraperitoneal bladder perforation that was first identified during a cystoscopy for bladder clot evacuation. This patient was at an increased risk of morbidity from an abdominal exploratory laparotomy given his poor performance status, history of prior abdominal surgeries and pelvic radiation, and multiple prolonged hospitalizations over the past six months. The accurate and rapid diagnosis of an intraperitoneal bladder perforation was deemed necessary prior to proceeding with an exploratory laparotomy. In this case, the initial identification of the bladder perforation occurred cystoscopically while the patient was under general anesthesia; hence, obtaining a multiplanar fluoroscopic cystogram was challenging. The intraoperative acquisition of a CT cystogram using a portable CBCT scanner allowed for the rapid radiographic determination of an intraperitoneal bladder perforation without having to transport a patient under general anesthesia.

Conventional management of an intraperitoneal bladder perforation includes laparotomy with its own associated morbidity. With the advent of laparoscopic surgical techniques, the laparoscopic repair of an isolated intraperitoneal bladder has been described, with an associated shorter hospital stay, faster return to functionality, and decreased morbidity [3]. Although laparoscopic repair of bladder perforations is less frequent, a diagnostic laparoscopy can replace the need to obtaining intraoperative cross-sectional imaging when a perforation is diagnosed cystoscopically. A laparoscopic approach would have been challenging in this patient given his prior history of pelvic radiation and abdominal surgeries.

The reported use of an intraoperative portable CBCT scanner in urologic surgeries is limited. Intraoperative CBCT scans were utilized by Zelefsky et al. to verify the positioning and dosimetry of prostate artery brachytherapy seeds [4]. The urologists were able to adjust the surgical plan and place additional seeds prior to anesthesia reversal based on the intraoperative CBCT scans obtained. The results were verified with a postoperative traditional CT scan, which demonstrated accurate correlation to the intraoperative CBCT scans. For patients with complex spinal anatomy and congenital malformations, intraoperative O-arm™ CBCT scans have been demonstrated by Hellström et al. to assist in surgical planning and navigate placement of an S3 stimulation electrode for sacral nerve stimulator lead implantation [5]. More recently, the use of a CBCT scan at the conclusion of a percutaneous nephrolithotomy was performed to evaluate for residual stone burden [6]. In a review of 19 cases, CBCT scans positively identified 9 patients with residual stone fragments that were removed by the urologist prior to anesthesia reversal. An eventual stone-free burden was confirmed with a postoperative noncontrast CT scan.

## 4. Conclusion

When an intraoperative bladder perforation is identified during cystoscopy, a single-view fluoroscopic cystogram may be inconclusive in differentiating between an intraperitoneal and extraperitoneal perforation. We describe the use of an O-arm™, a portable conical-beam CT scanner, to acquire an intraoperative CT cystogram to definitively diagnose an intraperitoneal bladder perforation and guide further management.

## Data Availability

Data are available on request.
